# Pharmaceutical prospects of Silymarin for the treatment of neurological patients: an updated insight

**DOI:** 10.3389/fnins.2023.1159806

**Published:** 2023-05-18

**Authors:** Shovit Ranjan, Akash Gautam

**Affiliations:** ^1^University Department of Zoology, Kolhan University, Chaibasa, Jharkhand, India; ^2^Center for Neural and Cognitive Sciences, University of Hyderabad, Hyderabad, India

**Keywords:** Silymarin, type 2 diabetes, neurological disorders, Alzheimer’s disease, Parkinson’s disease, antioxidant

## Abstract

**Background:**

Silymarin is a polyphenolic flavonoid complex extricated from dried fruits and seeds of the plant *Silybum marianum* L. Chemically, it is a mixture of flavonolignan complexes consisting of silybin, isosilybin, silychristin, silydianin, a minor quantity of taxifolin, and other polyphenolic compounds, which possess different bio medicinal values.

**Purpose:**

This review critically looks into the current status, pharmaceutical prospects and limitations of the clinical application of Silymarin for treating neurological disorders. In particular, Silymarin’s medicinal properties and molecular mechanisms are focused on providing a better-compiled understanding helpful in its neuro-pharmacological or therapeutic aspects.

**Methods:**

This review was compiled by the literature search done using three databases, i.e., PubMed (Medline), EMBASE and Science Direct, up to January 2023, using the keywords-Silymarin, neurological disorders, cognitive disorders, Type 2 Diabetes, pharmaceutical prospects and treatment. Then, potentially relevant publications and studies (matching the eligible criteria) were retrieved and selected to explain in this review using PRISMA 2020 (Preferred Reporting Items for Systematic Reviews and Meta-Analyses) study flow chart.

**Result:**

Since its discovery, it has been widely studied as a hepatoprotective drug for various liver disorders. However, in the last 10–15 years, several research studies have shown its putative neuroprotective nature against various brain disorders, including psychiatric, neurodegenerative, cognitive, metabolic and other neurological disorders. The main underlying neuroprotective mechanisms in preventing and curing such disorders are the antioxidant, anti-inflammatory, anti-apoptotic, pro-neurotrophic and pro-estrogenic nature of the bioactive molecules.

**Conclusion:**

This review provides a lucid summary of the well-studied neuroprotective effects of Silymarin, its underlying molecular mechanisms and current limitations for its usage during neurological disorders. Finally, we have suggested a future course of action for developing it as a novel herbal drug for the treatment of brain diseases.

## Neurological disorders

Neurological disorders (NDs) are nervous system diseases that mainly affect the brain, spinal cord, and nerves throughout the human body (Khanna et al., 2008). World Health Organization (WHO) data suggest that NDs are primary and essential global causes of mortality and morbidity (Mathers et al., 2003). The main NDs include Alzheimer’s disease (AD), Parkinson’s Disease (PD), Cerebral ischemia (CI), Multiple sclerosis (MS), Huntington’s Disease (HD), Meningitis, Epilepsy, and Stroke (Haddadi et al., 2020b). Better health care and increasing life expectancy in many countries will lead to more patients suffering from NDs. The studies on these disorders’ social and economic impact present a grim picture even for most developed nations (DiLuca and Olesen, 2014; Wynford-Thomas and Robertson, 2017). Therefore, detecting these NDs very early is necessary by using suitable biomarkers and designing appropriate preventive therapies or treatment protocols (Khanna et al., 2008). Unfortunately, most NDs remain incurable even after the rapid advancements in neuroscience, pharmacology and medical technology. Modern-day physicians cannot treat these NDs, and patients remain dependent on physiotherapists or rehabilitation providers. In order to shed insight into the pathophysiology of various brain disorders and aid in the development of targeted therapeutic strategies, more research is needed in these domains of study.

Some of the significant reasons which make NDs incurable are given here. Neurodegenerative disorders are a category of NDs in which progressive damage and death of neurons in the brain regions, like AD and PD, get diagnosed very late (Agrawal and Biswas, 2015). The precipitation of behavioral changes at an advanced stage means most neurons are already dead to an extent where recovery is impossible. Hence, patients will not respond to any medicine. Secondly, specific NDs are challenging to diagnose and differentiate from each other because of their similar symptoms and cognitive impairment. To make it worse, non-neurological symptoms can also precipitate very similar to neurological diseases. Due to their multifactorial and complex progression, brain disorders still need a complete understanding of the molecular pathways to develop specific therapeutic targets (Agrawal and Biswas, 2015). Further, even though few *in vitro*-tested drugs are available for the treatment of neurological disorders, these drugs show poor bioavailability inside the brain because of various biological obstructions, specifically the blood–brain barrier (BBB). Lastly, these drugs are costly and always come up with side effects. The drugs like neuroleptics, opiates and analgesics provide some relief to the NDs patients, but still, they need counselling and cognitive behavior therapy post-treatment (Gautam, 2022). Therefore, a practical pharmacotherapeutic approach for these NDs is required to reduce morbidity and mortality.

## Silymarin: a basic introduction

**Figure 1:**
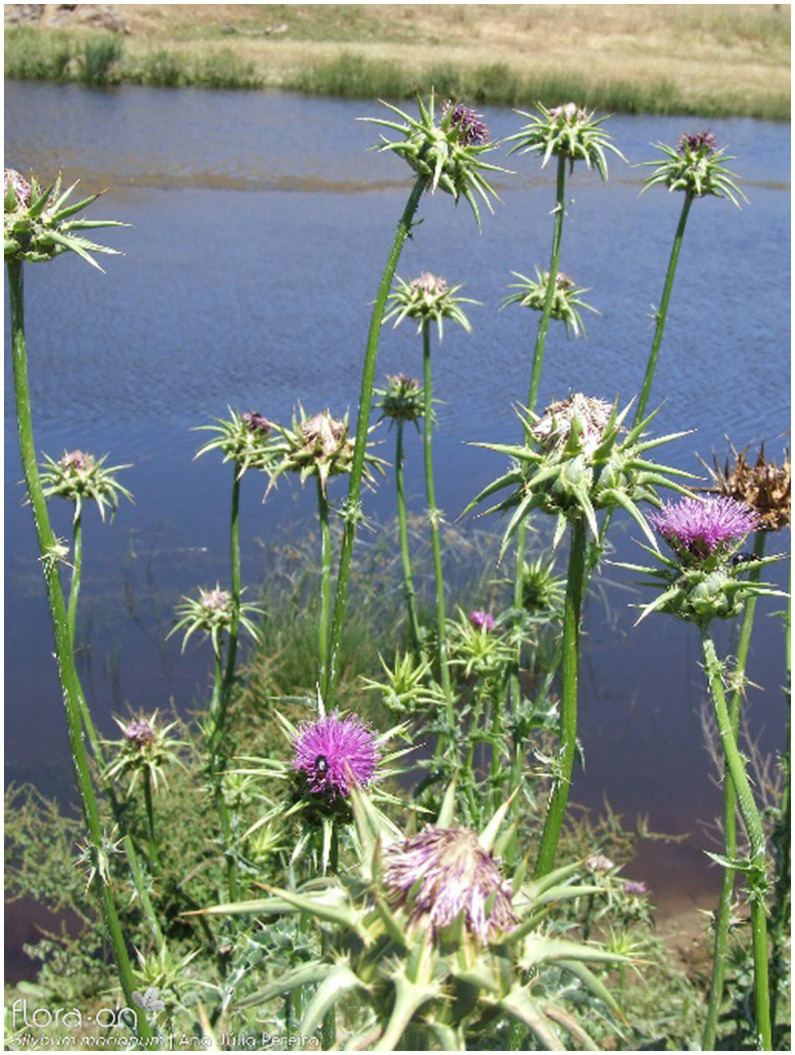
The whole plant of *Silybum marianum* L. (Image source: Ana Júlia Pereira, https://flora-on.pt/#/h7Tex, CC-BY-NC).

Keeping in view of these limitations, medical practitioners and patients always look for more holistic ways to treat brain disorders. In this regard, the non-allopathic traditional or complementary medicine system, which includes treatment through several herbs, metals, minerals, massages, exercises, and diet controls, has recently gained recognition (Ogut et al., 2019, 2022a,b; Guzelad et al., 2021; Li et al., 2021). *Silybum marianum* L. is a popular traditional Ayurvedic remedy for treating heartburn and its accompanying health problems. In recent years, this plant has also been studied for its beneficial properties for different NDs. Due to the milky streaks on its leaves, *Silybum marianum* is also known as milk thistle, Mediterranean thistle, blessed thistle, or scotch thistle (Scott Luper, 1998; Petrie et al., 2015). It is a tall, sturdy, biennial herb with a red or purple corolla, strong spinescent stems, pale green leaves and large purple flowering heads (Figure 1). It is mainly farmed for medicinal uses, although it has also been studied as a food source. Though it is an indigenous plant of the Mediterranean region of Europe, this is widely found in Kashmir, Southern and Western Europe, and Southern and North America these days (Pepping, 1999). It thrives well in sunny, warm ruderal meadows but avoids dry, stony soils.

Silymarin, a polyphenolic flavonoid compound, is isolated from dried fruits and seeds of *Silybum marianum* L. Chemically, it is a mixture of flavonolignan complexes consisting of silybin (i.e., silybin A and silybin B), isosilybin (i.e., isosilybin A and isosilybin B), silychristin, silydianin, a minor quantity of taxifolin, fatty acids and remaining polyphenolic compounds (Wagner et al., 1974; Rainone, 2005; Petrie et al., 2015). Out of all the constituents of Silymarin, approximately 60–70% of the mixture is silybin, which is also found to be accountable for the antioxidant function of Silymarin (Haddadi et al., 2020b). The present review provides a comprehensive summary of various research studies done to establish the beneficial effects of Silymarin on all major NDs along with its neuro-pharmacological or therapeutic aspects.

**Figure 2:**
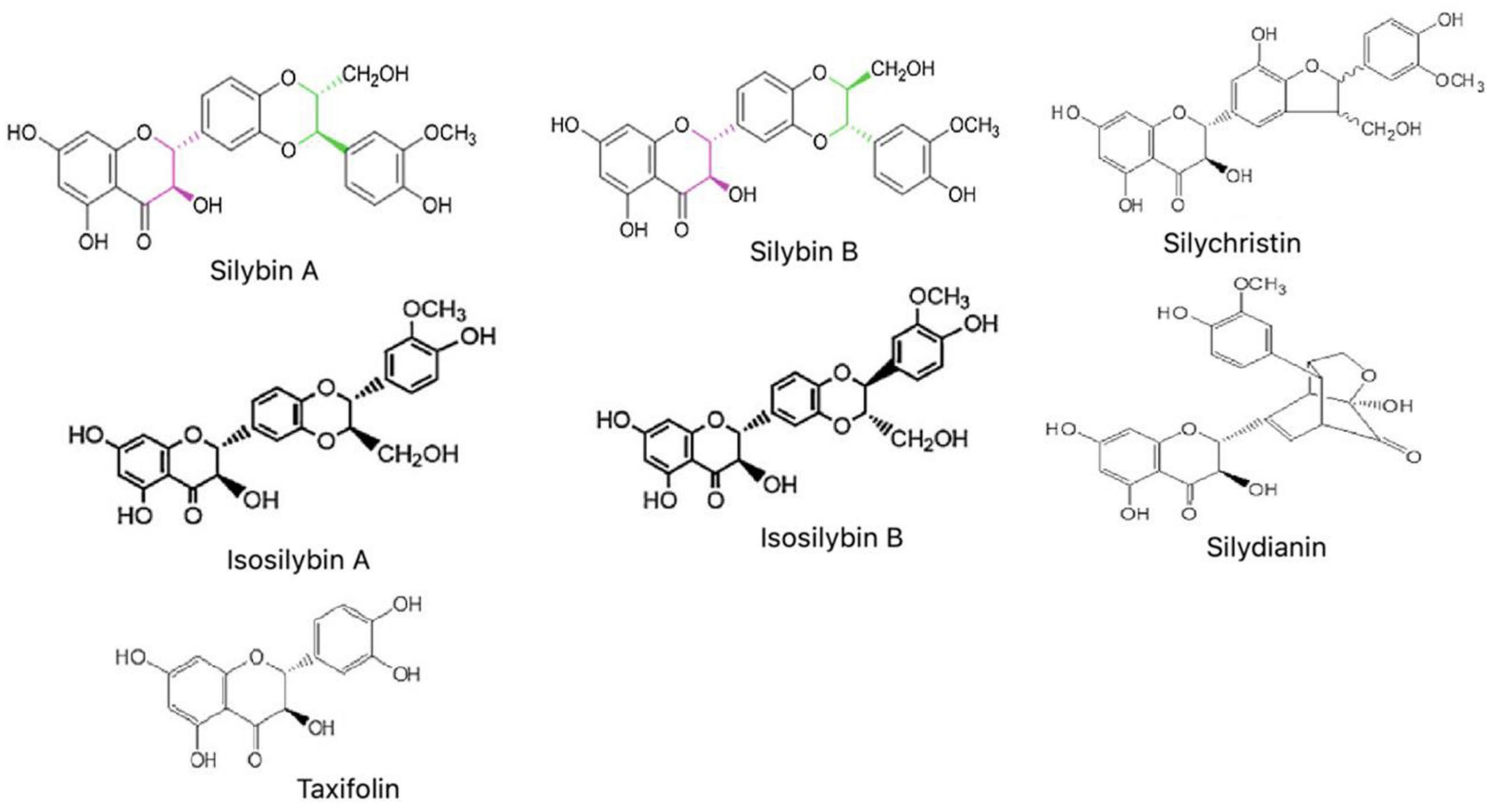
Chemical structures of main constituents of Silymarin (Silybin, Isosilybin, Silychristin, Silydianin, and Taxifolin).

The various parts of *S. marianum* are used to prepare the different extracts, composed of a mixture of distinct bioactive constituents. The chemical structure of all the main constituents is shown in Figure 2. Silibinin (or Silybin) A and B are the main biologically active component, capable of treating cancers, skin ailments and liver cirrhosis (Petrie et al., 2015; Neha and Singh, 2016). Silychristin is the next most copious flavonoid next silybin in the extracts of Silymarin, which has also presented antioxidant activity and non-cytotoxic effect against the different cancer cell lines (Biedermann et al., 2016). Isosilybin A and B have shown anti-prostate cancer activity by executing cell cycle arrest and cell apoptosis (Deep et al., 2007). Taxifolin has emerged as a unique bioactive flavonoid, showing promising inhibitory effects against oxidative stress, inflammation, hyperglycemia, AD, various malignancies, microbial infection, liver, cardiovascular and pulmonary disease (Sunil and Xu, 2019; Das et al., 2021). In-silico analysis on Silydianin has identified it as a novel and potent candidate in controlling COVID-19 disease through its inhibitory action on severe acute respiratory syndrome coronavirus 2 (SARS-CoV-2) spike protein. However, this claim needs to be validated by further *in vitro* and *in vivo* studies (Kousar et al., 2020; Majeed et al., 2021).

## Methods

**Figure 3:**
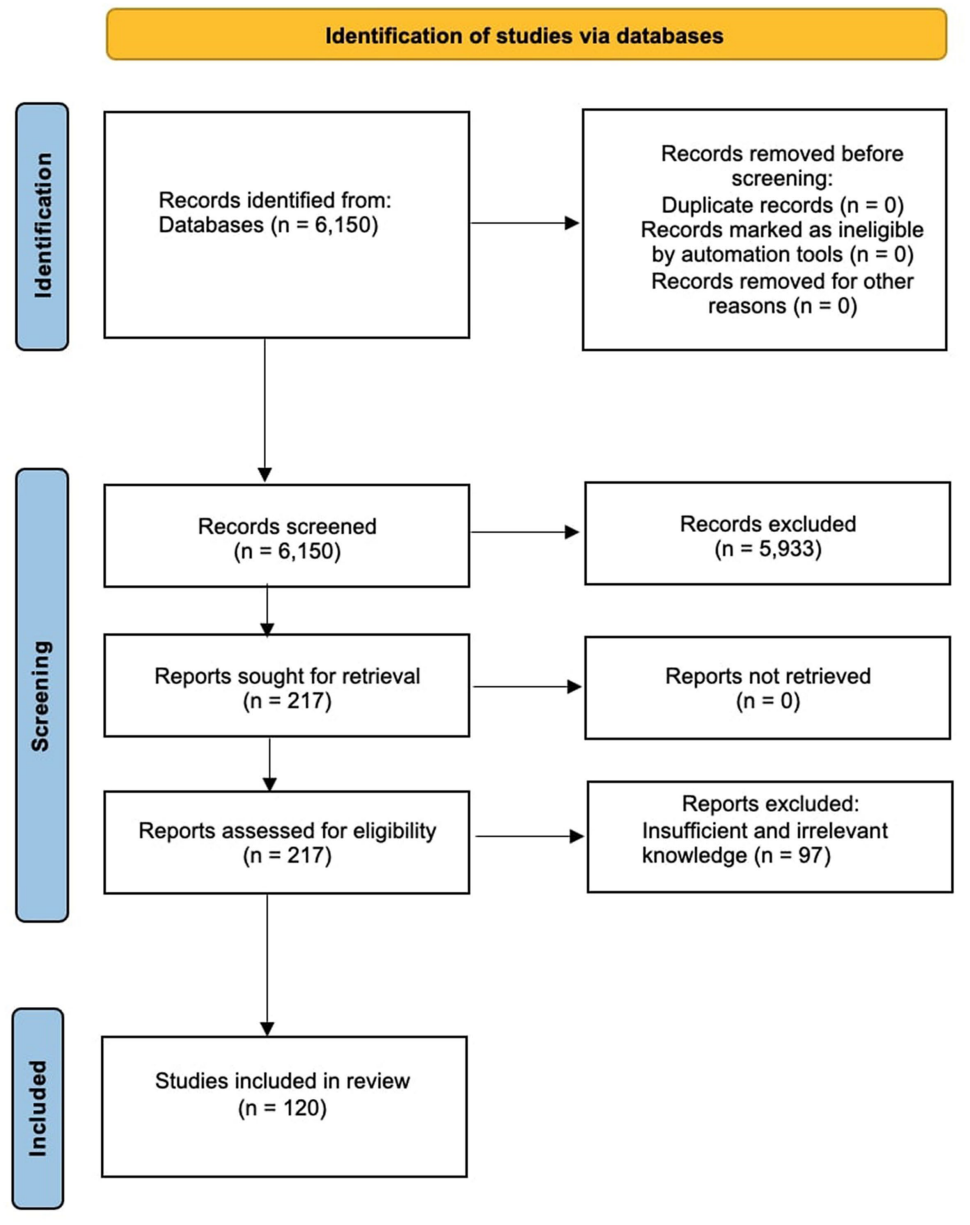
PRISMA 2020 study flow chart showing the number of included and excluded articles for this systematic review.

The literature search compiled this review to recognize the studies of Silymarin’s pharmaceutical prospects for treating NDs. Search keywords included “Silymarin,” “Silibinin,” “neurological disorders,” “Parkinson’s disease,” “Alzheimer’s disease,” “Cerebral Ischemia,” “cognitive disorders,” “pharmaceutical prospects,” and treatment.” Electronic databases, including PubMed, EMBASE, Science Direct, Scopus, Web of Science, Crossref and Medline, were searched with the terms’ Pharmaceutical prospects of Silymarin/Silibinin/silybin for the treatment’ in the title/abstract and ‘Neurological disease’, ‘Parkinson’s disease’, ‘Alzheimer’s disease’, ‘Cerebral Ischemia’, and ‘Cognitive disorder’ were searched in the whole text. Results were collected using only the articles in the English language up to January 2023. The primary exclusion criteria of articles depended on the title and abstract. Pharmaceutical prospects of Silymarin in some cases, in which the treatment was not related to NDs, were also excluded because the aim of the current review is to discuss pharmacological prospects and various mechanisms of action of this compound against neurological patients especially. After following the inclusion and exclusion criteria, 120 articles were retrieved and selected to explain in this review. The study design diagram has been demonstrated in Figure 3.

### Pharmacological properties of Silymarin

Silymarin has been chiefly known for hepatoprotective activities for a long time (Wang et al., 2015; AbouZid et al., 2016). Studies involving different animal models and human samples establish the prospective of its different extracts for antidiabetic, renal protective and anti-cancer activities (Wang et al., 2002; Lu et al., 2009a,b; Baluchnejadmojarad et al., 2010a,b; Murata et al., 2010; Karimi et al., 2011; Yin et al., 2011). Recent research shows that it has a neuroprotective role too in different NDs, such as AD, PD, epilepsy, CI and MS. These neuroprotective effects of Silymarin are attributed to its anti-inflammatory, antioxidant, anti-cancer, cardio-protective, radioprotective and anti-apoptotic activities in the biological systems, including model organisms and cell lines (Borah et al., 2013; Zholobenko and Modriansky, 2014; Ullah and Khan, 2018; Latacela et al., 2023).

Silymarin has been found to have no adverse side effects and has good safety, even when used in high doses (Saller et al., 2001; Duan et al., 2015). The approximate lethal dose 50 (LD50) values of Silymarin are 400, 385, and 140 mg/kg in mice, rats, and rabbits/dogs, respectively (Fraschini et al., 2002). One of its critical physiochemical properties, which makes it essential for its medicinal value, is that it is rich in oil and fatty acids (Khan et al., 2007; Neha and Singh, 2016). Because of poor solubility in the aqueous medium, Silymarin has also been found to have low bioavailability (Yang et al., 2013). However, in recent studies, different strategies are available to increase the same (Javed et al., 2011). For example, it is usually administered in capsule form with different bioactive formulations in both *in vitro* and *in vivo* animal models. It is marketed as capsules and tablets by Liverpool, Silipide and Legalon (Kaur and Agarwal, 2007; Karimi et al., 2011). The half-life of Silymarin, which is eliminated in bile, is 6–8 h. (Morazzoni et al., 1993; Petrie et al., 2015).

As per the data from pharmacological studies, it is a safe herbal medicine. However, adverse effects like headaches, gastroenteritis and dermatological symptoms are also reported in some cases of improper administration of therapeutic dosages in clinical trials (Křen and Walterová, 2005; Karimi et al., 2011). On the other hand, concurrent use of Silymarin with a P-glycoprotein (P-gp) substrate drug has been found to increase its cellular distribution across the blood–brain barrier (BBB). P-gp is the most commonly known transporter protein in BBB, which inhibits the efflux of substrate drugs into the brain, thus establishing itself as a critical factor in improving the bioavailability of various drugs in patients with a neurological disorder (Ravikumar Reddy et al., 2016). Silymarin can alter the pharmacokinetics of different allopathic drugs when co-administered in rodent models of NDs. The multiple pharmacological actions of Silymarin can thus be used synergistically for efficacious therapy in such cases (Ghosh et al., 2010).

### Silymarin for psychiatric disorders

Psychiatric disorders are a broad range of mental health abnormalities that causes significant disturbance in mood, thinking and behavior (Hofmann, 2014). The psychiatric disorders prevalent across the globe include anxiety, depression, schizophrenia, mood disorders, and obsessive–compulsive disorders (Gazzaniga and Heatherton, 2006). These disorders are linked with the increased probability of developing other major NDs (Jacob et al., 2010; Byers and Yaffe, 2011). Silymarin has been found to have a vital role in the treatment of such psychiatric disorders through different mechanisms (Grant and Odlaug, 2015; Khoshnoodi et al., 2015; Thakare et al., 2017, 2018; El-Elimat et al., 2018; Kosari-Nasab et al., 2018; Yön et al., 2019; Tabaa et al., 2022). In the forced swim and tail suspension tests, silymarin administration significantly decreased the immobility period in mice, indicating that it may have antidepressant effects (Bansal et al., 2013; Khoshnoodi et al., 2015; Thakare et al., 2016). In another study by El-Elimat et al. (2019), Silymarin was found to have an anxiolytic effect as assessed through the elevated plus maze and open field tests in rat models.

Several brain areas, like the hippocampus and frontal cortex, show a noticeable reduction of dopamine, serotonin and norepinephrine neurotransmitters during the depression in animal models (Tse and Bond, 2002; Nutt, 2008; Mao et al., 2011). *In vivo* neurochemical studies have shown that Silymarin can restore the levels of these neurotransmitters in the brain centers (hippocampus and cerebral cortex) of rodents (Osuchowski et al., 2004; Thakare et al., 2017; Kosari-Nasab et al., 2018; Thakare et al., 2018). Silymarin has also been found to restore the normal levels of antioxidants catalase and glutathione along with a reduction in malondialdehyde formation in the above said brain centers of the depression rodent models, thus suggesting the role of Silymarin as an antidepressant (Thakare et al., 2017). In another study by Grant and Odlaug (2015), Silymarin inhibited free radicals and nitric oxide formation in humans with obsessive–compulsive behaviors. Furthermore, neurotrophic factor BDNF level was found to be increased in the above-said brain regions after Silymarin treatment in rodents’ model of depression, suggesting BDNF is one of the crucial targets for antidepressant action of Silymarin (Thakare et al., 2017; Kosari-Nasab et al., 2018; Thakare et al., 2018).

Inflammation and psychiatric disorders are closely intertwined (Toklu et al., 2008; Morishima et al., 2010; Nazemian et al., 2010; Gharagozloo et al., 2013a; Al-Drees and Khalil, 2016). Dysregulation of pro- and anti-inflammatory signaling pathways due to different stressors during psychiatric disorders leads to immunologically modulated neuroinflammation, thus showing the anti-inflammatory property of Silymarin in several *in vivo* and *in vitro* studies (Toklu et al., 2008; Morishima et al., 2010; Nazemian et al., 2010; Gharagozloo et al., 2013a; Al-Drees and Khalil, 2016). Silymarin decreases the levels of the two most prominent pro-inflammatory cytokines, interleukin 6 (IL-6) and tumor necrosis factor-alpha (TNF-α), suggesting its potential anxiolytic and antidepressant activity. Furthermore, Silymarin administration has been shown to decrease the corticosterone levels in various animal models of depression in different studies (Thakare et al., 2017; Kosari-Nasab et al., 2018; Thakare et al., 2018), hence correlating antidepressant activity of Silymarin to its effects on hypothalamic–pituitary–adrenal axis regulation.

### Silymarin for neurodegenerative disorders

Neurodegenerative disorders (NDDs) occur through the gradual and progressive degeneration of nerve cells in the central or peripheral nervous system and, ultimately, the death of these neurons. AD, HD, PD and amyotrophic lateral sclerosis are the most common NDDs, accounting for the principal causes of death across the world (Ranjan and Sharma, 2018; Ranjan, 2020; Pereira et al., 2021). The probability of the onset of these diseases increases with age (Wyss-Coray, 2016). The neuroprotective potential of Silymarin is well-explored and reported in animal and cellular models of NDDs (Wang et al., 2002; Lu et al., 2009b; Baluchnejadmojarad et al., 2010b; Murata et al., 2010; Yin et al., 2011; Amato et al., 2019).

In the AD model of *Caenorhabditis elegans*, Kumar et al. (2015) found that the Silymarin treatment reduces amyloid b-protein in muscle tissues by increasing resistance to oxidative stress. Further behavioral study on APP transgenic mice showed that the treatment with Silymarin decreases AD-like phenotypes and improves AD-induced behavioral anomalies (Murata et al., 2010). In another study, Silymarin treatment on SH-SY5Y neuroblastoma cell lines resulted in the inhibition of amyloid β aggregation as well as hydrogen peroxide production in the cell, thus protecting it from the impairment caused by amyloid β-induced oxidative stress (Yin et al., 2011). The main mechanisms regarding the protective effect of Silymarin against AD neurodegeneration are the prevention of amyloid β plaque aggregation by their disintegration and suppression of APP expression (Murata et al., 2010; Murakami et al., 2013; Yaghmaei et al., 2014); protection of dopaminergic neurons through inhibition of microglia activation, inflammation, and apoptosis (Wang et al., 2002, 2012); downregulation of acetylcholinesterase activity (Duan et al., 2015; Aboelwafa et al., 2020); upregulation of neurotrophic factors and mitigation of autophagy, oxidative stress, and apoptosis (Raza et al., 2011; Song et al., 2017).

A reduction in oxidative stress by Silymarin is linked with a decline in reactive oxygen species level, lipid peroxidation, and nitric oxide generation (Lu et al., 2009a; Baluchnejadmojarad et al., 2010b; Yin et al., 2011), as well as upregulation of the enzymatic rate of different antioxidant enzymes (reduced glutathione, catalase and superoxide dismutase; Nencini et al., 2007; Lu et al., 2009b; Kumar et al., 2015; El-Marasy et al., 2018; Ali et al., 2019; Aboelwafa et al., 2020; Singh et al., 2020). Apart from antioxidant properties, Silymarin treatment has shown anti-inflammatory action in AD models by suppressing toll-like receptor 4 (TLR4) pathways and decreasing the increased mRNA levels of TNF-α, IL-1β and NF-κB (Ali et al., 2019; Aboelwafa et al., 2020). Silymarin has also exhibited estrogen-like activity through selective activation of ER-β (Seidlova-Wuttke et al., 2003; Plíšková et al., 2005). Recently, the regulative effects of Silymarin treatment on the relative abundance of essential gut microbiota involved in AD development have also been studied in transgenic APP/PS1 mice (Shen et al., 2019). The beneficial effects of Silymarin based on the gut-brain axis have been discussed below in the subsequent headings.

Similarly, the neuroprotective roles of Silymarin in different PD models have been observed at behavioral and cellular levels. Some of the critical observations are improvement in catalepsy and motor impairment (Haddadi et al., 2015), attenuation of increased myeloperoxidase activity (Haddadi et al., 2013), reduction of nitrite content and lipid peroxidation (Baluchnejadmojarad et al., 2010b), the elevation of antioxidant activity of enzymes like catalase, glutathione peroxidase, superoxide dismutase and glutathione reductase (Haddadi et al., 2020b), reduction of pro-inflammatory cytokines IL-6 and TNF-α in CSF (Haddadi et al., 2013), and execution of anti-apoptotic effects by down-regulation of caspase-3, Bcl-2, Bax protein (Haddadi et al., 2018). The dopaminergic neurons are rescued from neurodegeneration by Silymarin by modifying apoptotic pathways leading to the increasing death of apoptotic cells (Pérez-H et al., 2014). Silymarin treatment reduces CYP2E1, Bax, phosphorylated: unphosphorylated p53, GSTA4-4, VMAT2 and Caspase 9 gene expression in PD models (Singhal et al., 2011). In lipopolysaccharide animal models for PD, Silymarin also prevents dopaminergic neuronal loss by blocking microglia stimulation and NF-κB synthesis or through the suppression of major cytokines levels and nitric oxide levels after iNOS production (Wang et al., 2002).

However, the neuroprotective results of Silymarin are lacking in the case of other NDDs (like HD and amyotrophic lateral sclerosis), which can be beneficial additions to the above findings in future for a better understanding of the Silymarin effects on NDDs.

### Silymarin for other neurological and cognitive disorders

Few studies of Silymarin in the case of brain ageing and brain tumors in animal models are available. In a study by Galhardi et al., 2009, Silymarin treatment ameliorates oxidative stress by decreasing the level of oxidized proteins in the aged brain, thus preventing age-linked pathological deteriorative processes in the brain. Silymarin has also shown neuroprotective effects in different induced brain injury models by preventing oxidative damage (Nencini et al., 2007; Toklu et al., 2008).

Silymarin treatment is well-studied in the case of rodent models of cerebral ischemia. The Silymarin-induced neuroprotection during CI is attributed to its antioxidant, anti-inflammatory and anti-apoptotic responses (Hou et al., 2010; Raza et al., 2011; Moghaddam et al., 2020). Silymarin reduces oxidative stress by inhibiting the production of reactive oxygen species, inducible nitric oxide synthase and myeloperoxidase (Hou et al., 2010). At the same time, it increases the level of antioxidant enzymes like superoxide dismutase, catalase, glutathione peroxidase, glutathione reductase, and glutathione (Moghaddam et al., 2020). Silymarin exhibits an anti-inflammatory response in CI damage by suppressing the activation of TNF-β, NF-κB, STAT-1, COX-2 and intrusion in leukocytes. These changes lead to a considerable reduction in brain cell death and necrotic tissue volume, improvement in memory loss and psychomotor behavior in the ischemic rat model (Muley et al., 2012, 2013). Other research has found that Silymarin medication reduces brain damage (Karabag and Koçarslan, 2020), brain swelling and lesion volume, the severity of the cognitive disorder and motor deficits (Gupta and Gupta, 2017), and TNF- and IL-6 mRNA expression (Kosari-Nasab et al., 2018; Moghaddam et al., 2020) in ischemic animal models. Silymarin downregulates several apoptotic-inducing molecules like p53, Caspase-3 and 9, and Apaf-1; and triggers the intrinsic pathway of apoptosis by preventing the apoptosome formation in the ischemic rat model (Raza et al., 2011). The activation of the Akt/mTOR signaling pathway, downregulation of the inflammatory indicator protein (NF-κB) and upregulation of the anti-apoptotic indicator protein (Bcl-2) in the CI brain are other mechanisms of neuroprotection conferred by Silybinin, one of the biologically active ingredients of Silymarin (Wang et al., 2012). Furthermore, Silymarin works as a neuroprotective drug in intracerebral hemorrhage by downregulating the expression of NLRP3 and p65 (components of NF-κB), the inflammasome-mediated caspase-1/IL-1 production and upregulating the Nrf-2/HO-1 signaling in mice model (Yuan et al., 2017).

Multiple sclerosis is the most frequent immune-mediated neurological disorder, where neuronal demyelination causes abrupt signal transmission (Huang et al., 2017). The effects of Silymarin on MS are also studied in different *in vitro* and clinical studies (Navabi et al., 2019; Shariati et al., 2019; Abbasirad et al., 2021; Ghiasian et al., 2021). Silymarin has shown hepatoprotective effects and an antioxidant role in MS by decreasing the oxidative stress biomarkers, increasing the antioxidant enzyme level, and acting as a scavenger of free radicals (Ghiasian et al., 2021). *In vitro* studies and clinical trial also shows immunoregulatory, i.e., immunosuppressive effects on inflammatory responses in MS *via* the regulation of T-helper cells (Th1, Th17). It suppresses the proliferating activity of Th1 and Th17 and inhibits the mRNA level of Th1’s specific transcription factor (T-bet) and interferon-gamma synthesis by these cells (Navabi et al., 2019; Shariati et al., 2019; Abbasirad et al., 2021). It has also been found to be involved in increasing or restoring the regulatory T-cells (Treg) function in MS, most probably through activation of JAK3/STAT5 signaling, thus resulting in anti-inflammatory and immunomodulatory effects (Shariati et al., 2019; Abbasirad et al., 2021). These inhibitory effects of Silymarin on T-cell response have supported its application for the treatment of patients of autoimmune diseases, including MS (Gharagozloo et al., 2010, 2013b,c).

However, in the case of epilepsy, Silymarin does not show neuroprotective effects. In a study, it has been found that Silymarin pre-treatment was unable to protect and rescue the neurons of hippocampal regions against neurotoxicity in epileptic models (Sedaghat et al., 2017).

Silymarin’s role in cognitive impairments is also well-studied in animal studies (Reid et al., 1999; Lu et al., 2010; Neha et al., 2014; Yaghmaei et al., 2014; Yön et al., 2019). A study by Reid et al. (1999) showed that the Silymarin treatment improves ethanol-induced learning deficits in rats. In another study, Silymarin supplementation improved learning and memory in diabetes-induced cognitively impaired rats by elevating BDNF levels (Yön et al., 2019). Moreover, Silymarin administration has also shown significant improvement in memory function in high-fat diet-induced dementia and the amyloid b-induced Alzheimer’s model in mice and rats, respectively (Neha et al., 2014; Yaghmaei et al., 2014). Furthermore, the effect of silibinin on cognitive impairment is also associated with the ameliorative effect by decreasing the dopamine and serotonin values in the prefrontal cortex and hippocampus regions of the brain, respectively, in mice models (Lu et al., 2010).

### Indirect effects of Silymarin through the gut–brain axis

Many studies have stated that Silymarin has low bioavailability because of poor water solubility (Dixit et al., 2007; Javed et al., 2011; Sornsuvit et al., 2018; Di Costanzo and Angelico, 2019; Kesharwani et al., 2020). Silymarin intake through the oral route undergoes two-stage biotransformation, i.e., stages I and II. The rapid and complete biotransformation of silybin during stage II and its absorption in the intestinal epithelium is the primary reason for its low bioavailability. However, it has been found that efflux transporters multidrug resistance-associated protein 2 (MRP2) and breast cancer resistance protein (BCRP) on the apical side of the intestinal epithelium also play a crucial role in further affecting the Silybin absorption and excretion, accounting for its low bioavailability (Xie et al., 2019). Few studies on cell lines and animal models have shown that the inhibitors of these two transporter proteins, Tangeretin, can augment Silybin absorption (Yuan et al., 2018; Xie et al., 2019).

The excretion of Silymarin involves rapid elimination of both free and compound forms *in vivo*. It has been discovered that urinary excretion of silybin is low, accounting for just 1–2% of an initial oral dosage over 24 h; however, hepatobiliary clearance is considerable, indicating the substantial involvement of the MRP2 efflux transporter (Miranda et al., 2008; Xie et al., 2019). The role of another efflux transporter P-gp (P-glycoprotein multidrug transporter), is also very well documented in various studies that hinder the entry of various therapeutic agents, including Silymarin, into the brain across BBB. The concurrent use of Silymarin with P-gp substrate and nanoformulations-based P-gp trafficking approaches (like nanocarriers) are reported in many studies for easing the drug transport across BBB (Reddy et al., 2016; Pathan and Shende, 2021).

The use of inhibitor Tangeretin against these efflux transporters is also reported to decrease silybin’s biliary excretion index and biliary clearance *in vitro*. Therefore, in the long run, these inhibitors of efflux transporters, which are accountable for the absorption and excretion of silybin and its metabolites, can enhance the bioavailability, bioactivity and pharmacological effects of silybin *in vivo* (Yuan et al., 2018; Xie et al., 2019).

The role of Silymarin is also reported in other important metabolic disorders like obesity, diabetes, and fatty liver disease in different animal and human studies (Tajmohammadi et al., 2018). In a study by Qin et al. (2017), it was found that seed extracts of this medicinal plant have potential antidiabetic effects. Silymarin treatment in rats is protective against high-fat diet-induced metabolic disorders like obesity, hyperlipidemia, type 2 diabetes (T2D), and hepatopathy. These beneficial effects of Silymarin are a result of the significant reduction in high-sensitive C-reactive protein of serum, triglycerides, low-density lipoprotein cholesterol, total cholesterol, alanine transaminase, gamma-glutamyl transferase and insulin levels, thus indicating the possibility of using Silymarin as an effective supplement for the improvement of insulin and leptin sensitivity in these disorders (Sayin et al., 2016). In another study, Silymarin was found to reverse metabolic damage in a diabetic rat model by lowering blood glucose levels, cholesterol, lipoperoxidation index and oxidative phosphorylation abnormalities in the liver’s mitochondria (Vengerovskii et al., 2007). Silymarin has also been shown to improve liver damage and insulin resistance by decreasing inflammatory response and plasma lipid levels in high-fat diet-induced obese and T2D mice (Guo et al., 2016). Arshad et al. (2022) also studied Silymarin’s protective role in controlling streptozotocin-induced T2D in rabbits. Not only in laboratory studies, but there are also pieces of evidence of Silymarin in clinical studies regarding the improvement of blood glucose levels and lipid profiles in T2D patients (Huseini et al., 2006; Pereira et al., 2016; Zarvandi et al., 2017). The hypoglycemic effect of Silymarin in a diabetic rat model in the treatment and prevention of diabetic neuropathy is also well studied, as evidenced by the significant improvement in motor nerve conduction velocity, hyperalgesia, malondialdehyde level and antioxidant enzyme superoxide dismutase (Baluchnejadmojarad et al., 2010a; Mannelli et al., 2012, 2013).

Silibinin has also reduced insulin resistance in the nonalcoholic fatty liver disease (NAFLD) animal model (Chou et al., 2012; Salamone et al., 2012; Yao et al., 2013; Salomone et al., 2016; Zhong et al., 2017). The probable underlying mechanism for the same is the reduction of visceral obesity by a decrease in visceral fat, enhancement of lipolysis by upregulation of triglyceride lipase expression in adipose tissues and inhibition of gluconeogenesis by downregulation of associated genes (like forkhead box O1, glucose-6-phosphatase, PEP carboxykinase; Yao et al., 2013). More specifically, Silymarin and Silibinin have been studied to control oxidative stress in lipid metabolism associated with NAFLD and nonalcoholic steatohepatitis (Salamone et al., 2012; Salomone et al., 2016). In a meta-analysis study by Zhong et al. (2017), the therapeutic effect of Silymarin during NAFLD has been attributed to a considerable decrease in transaminase level. In another study, Silymarin has also been reported to alleviate the effects caused by long-term alcohol consumption in mice by reducing the liver size and total triglyceride levels to normal. It has also been found to restore normal histology in liver lobules, showing decreased accumulation of lipid drops, thus reducing liver damage to a great extent (Chou et al., 2012).

### Molecular mechanisms of neuroprotection by Silymarin

**Figure 4:**
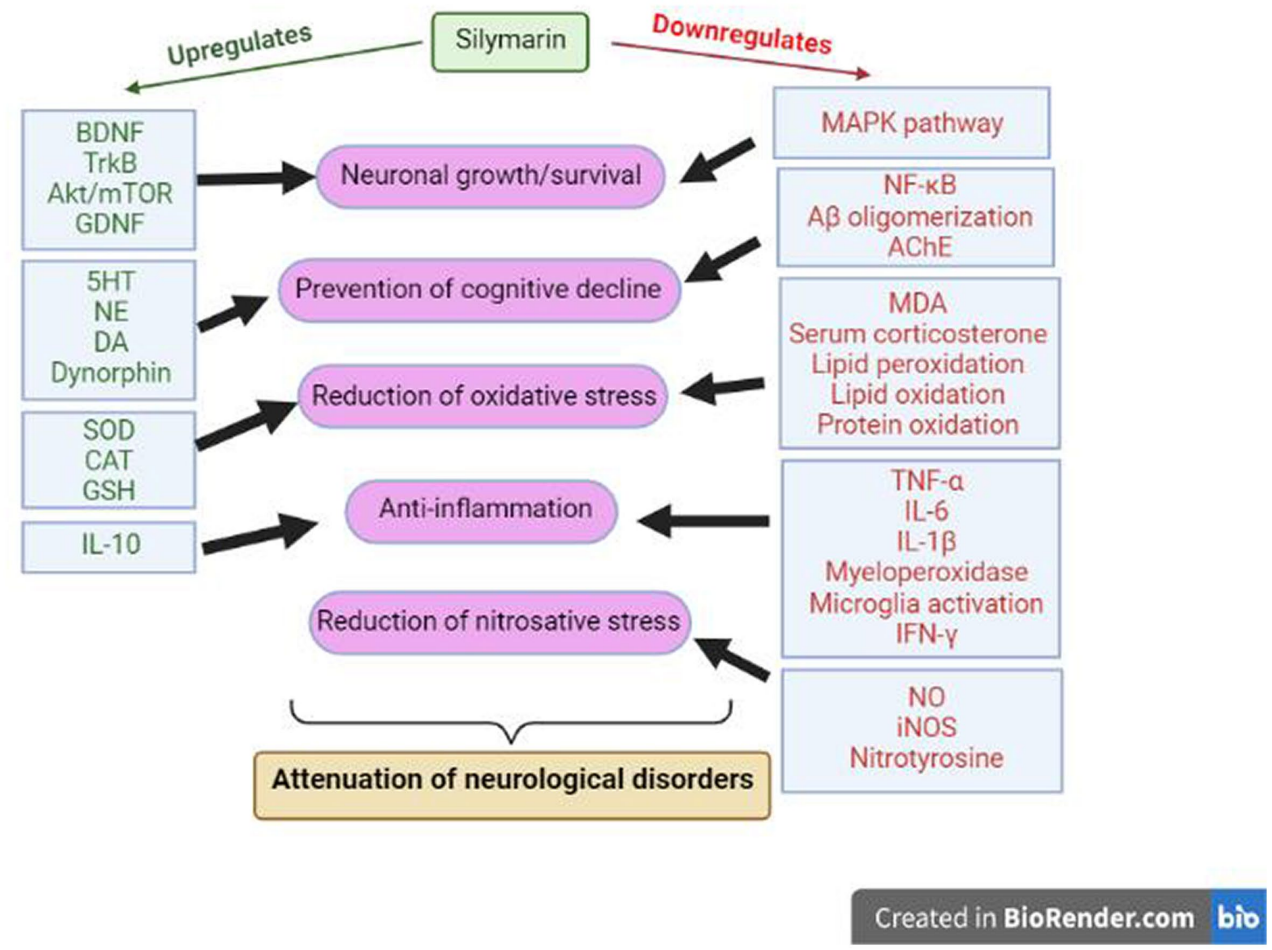
Molecular mechanisms of Silymarin during neuroprotection. BDNF: Brain Derived Neurotrophic Factor, TrkB: Tropomyosin receptor kinase B, GDNF: Glial cell line-derived neurotrophic factor, NE: Norepinephrine, DA: Dopamine, SOD: Superoxide dismutase, CAT: Catalase, GSH: Reduced L-glutathione, IL: Interleukin, TNF: Tumor necrosis factor, IFN: Interferons, MDA: malondialdehyde, NO: nitric oxide, iNOS: Inducible nitric oxide synthase, NFκB: Nuclear factor kappa B, AChE: Acetylcholinesterase.

The various mechanisms by which one can explain this neuroprotective effect of Silymarin in different models of brain disorders are linked to the regulation of neurotransmitters (Duan et al., 2015), inhibition of oxidative stress in the brain (Galhardi et al., 2009; Lu et al., 2009b), inhibition of the inflammatory reaction associated with neurodegeneration (Wang et al., 2012), regulation of neurotrophic factors (Song et al., 2017), mimicking of estrogenic activity (Kummer et al., 2001; Seidlova-Wuttke et al., 2003) and inhibition of cellular apoptotic machinery (Raza et al., 2011). The result of several tightly regulated neuronal pathways is the prevention of β-amyloid aggregation, neutrophil inhibition, regulation of inflammatory mediators, restoration of excitatory neurotransmitters, and neuronal survival, finally leading to normal cognitive functions (Figure 4).

The neuroprotective effects executed by Silymarin may be due to the involvement of the BDNF/TrkB pathway. BDNF/TrkB is vital for the survival of the neurons, which generally get impacted during neurological disorders like anxiety and depression. Silibinin upregulates the expression of BDNF/TrkB and inhibits hippocampal neurons’ autophagy, hence ameliorating cognitive decline during neurological disorders (Song et al., 2017). Tabaa et al. (2022) have also shown the modulation of endogenous dynorphin (Dyn)/glial cell line-derived neurotrophic factor (GDNF) and mitogen-activated protein kinase (MAPK) pathway by Silymarin during neurological disorders. This study found that Silymarin increases interleukin 10 (IL-10), GDNF and Dyn levels, decreases IFN-γ, TNFα, and IL-1β levels, and down-regulated the MAPK pathway. In some studies, these neuroprotective effects of Silymarin are also reported to be correlated with its estrogen-like activity and potential to bind and activate ER-b (Kummer et al., 2001; Seidlova-Wuttke et al., 2003; Plíšková et al., 2005; Baluchnejadmojarad et al., 2010b). Estrogen is well known to possess neuroprotective activity through different pathways, *viz.* genomic, nongenomic and anti-inflammatory mechanisms.

In another study, the level of excitatory neurotransmitters (like serotonin, dopamine and norepinephrine), monoamines, BDNF, superoxide dismutase, and catalase are upregulated by the Silymarin, along with an inhibition of inflammatory pathways by decreasing the level of TNFα, IL-6, malondialdehyde formation, and serum corticosterone (Baluchnejadmojarad et al., 2010a,b; Kosari-Nasab et al., 2018; Thakare et al., 2018; Haddadi et al., 2020a; Moghaddam et al., 2020). These regulations again hint at the anti-inflammatory and antioxidant properties of Silymarin. Many anatomical changes are observable during neurological disorders like degeneration of pyramidal neurons, astrocytes and oligodendrocytes, deranged myelin coats, defective BBB, focal degranulation of myelin sheaths, that were found to be restored after the treatment with Silymarin in animal studies (Aboelwafa et al., 2020). Similarly, the reduced activity of glutathione, superoxide dismutase and catalase during neurological disorders is restored to a normal level by enhancing their activity significantly (Aboelwafa et al., 2020). Moreover, a decrease in the activity of lipid peroxidase, nitric oxide synthase, and acetylcholinesterase activity, as well as in the level of IL-1 β, prevents the aggregation of amyloid β, one of the major characteristic features of neurodegenerative disorders (Aboelwafa et al., 2020).

As the enteric and nervous systems are interconnected through the brain-gut axis, different biochemical signaling induced by the pharmacokinetics of Silymarin may affect the treatment of neurological disorders (Sayin et al., 2016). Silymarin has been found to impair the level of serum total cholesterol, low-density lipoprotein cholesterol (LDL-C), triglycerides, susceptible C-reactive protein, leptin, insulin, plasma transaminase and glucose during diabetes or insulin resistance (Sayin et al., 2016). On the other hand, lipolysis is enhanced by the upregulation of adipose triglyceride lipase, and gluconeogenesis is decreased by inhibition of forkhead box O1, PEP carboxykinase and glucose-6-phosphate expression level. An increase in lipolysis and inhibition of gluconeogenesis reduces insulin resistance and can attenuate the symptoms of neurological disorders (Yao et al., 2013).

Based on all the above findings, Silymarin can be a promising candidate drug for preventing brain disorders. Silymarin’s protective effects during NDs are precisely executed by improving the neuroplasticity and neurotransmission in these disorders (Yan et al., 2015).

### Challenges and future directions in its pharmaceutical usage

Silymarin’s therapeutic benefits in neurological disorders *via* diverse pathways have led to the challenge of utilizing it in the pharmaceutical sector. In other words, various molecular mechanisms by which Silymarin can show its neuroprotective properties still need to be precise. For example, the binding of Silymarin to ER-β receptor leads to cellular signaling similar to estrogen, modulation of Aβ oligomerization without affecting the activity of β-site Amyloid-precursor-protein Cleaving Enzyme (BACE) or cleavage of APP, the crossing of BBB by Silymarin etc. are some of the less explored avenues for the treatment of neurological disorders (Borah et al., 2013). Consequently, more research is needed to traverse these pathways and fully understand Silymarin-mediated neuroprotection.

However, Silymarin is considered very safe in different studies, and fewer reports on its adverse effect show allergic responses (Geier et al., 1990; Jacobs et al., 2002). Silymarin is found to be safe at therapeutic doses in humans. It can be well tolerated even at a high dose (700 mg 3 times a day for 168 days) with gastrointestinal discomforts like nausea and diarrhea in some cases. In one clinical study, Silymarin was also reported as safe in pregnancy, but more human studies are required to confirm the same. Silymarin has been studied to have no significant effects on the cytochrome P450 system. The non-toxic nature of Silymarin allows its urgent clinical evaluation for its potential use as a neuroprotective molecule in humans.

Silymarin side effects include moderate laxative effect, urticaria, nausea, abdominal discomfort, musculoskeletal pain, headache, and itchiness (Mayer et al., 2005). Despite its multiple advantages, silibinin and all other compounds found in Silymarin, especially silychristin, appear to be potent thyroid disruptors by blocking the monocarboxylate transporter 8 (MCT8). Long-term silymarin usage can induce thyroid problems, and Silymarin can cause the Allan-Herndon-Dudley syndrome if used during pregnancy. However, the study on the interactions of Silymarin with the drug is low, which is another challenge in its pharmaceutical usage (Soleimani et al., 2019).

Another challenge of using Silymarin in pharmaceuticals was the low solubility of the molecule, resulting in low oral bioavailability from the gastrointestinal tract. It has been shown in the study that Silymarin has not only low aqueous solubility, but lipophilic properties are also lacking in the molecule (Woo et al., 2007). However, strategies like using solid dispersions, self-micro emulsifying drug delivery systems, liposomes and porous silica nanoparticles have been explored recently to improve the solubility and bioavailability of Silymarin (Ghosh et al., 2010; Yang et al., 2015). The self-micro emulsifying drug delivery system has shown a 3.6-fold boost in bioavailability (Woo et al., 2007). Moreover, a solid dispersion system (using Silymarin, polyvinylpyrrolidone and Tween-80) has shown an increase in the solubility by about 650-times along with the physical and chemical strength of the molecule by 6 months (Hwang et al., 2014).

Furthermore, silymarin-loaded porous silica nanoparticles produced an initial rapid discharge followed by continuous release of the molecule over 72 h (Cao et al., 2012), and liposome-mediated silymarin administration produced superior outcomes in terms of solubility and bioavailability than silymarin solution (Kumar et al., 2014). Recently, an adequate formulation of Silymarin prepared using chitosan nanoparticles was used for treating cerebral ischemia/reperfusion injury in rats. The results showed more effective preventive effects *via* improvement in the bioavailability of the molecule (Moghaddam et al., 2020).

## Conclusion

The present review article summarizes the essential neuroprotective activities and therapeutic potential of the Silymarin molecule in different brain disorders, including neurodegenerative, psychiatric and cognitive disorders. The mode of action or mechanism by which this neuroprotective nature of Silymarin is exhibited is very diverse, ranging from general antioxidant, anti-apoptotic, and anti-inflammatory to specific anti -amyloidogenic and pro-estrogenic natures. These diverse neuroprotective mechanisms exhibited by this molecule in the brain hold great promise to be considered a new candidate for the biomedical treatment of brain disorders. The non-toxic nature gives an extra advantage to Silymarin as a holistic medication for NDs. However, further research on its low aqueous solubility and bioavailability in the brain must be taken care of to prepare the molecule for any final clinical trials of such brain disorders. More human trials with Silymarin treatment, knowledge of precise molecular mechanisms of neurological disorders and integration of nanotechnology during the synthesis and delivery of Silymarin nanoformulations can help us to utilize the maximum benefit of this promising pharmacological agent for the treatment of neurological disorders in future.

## Author contributions

SR: literature review, data analysis, and writing—figures and original draft. AG: conceptualization, writing—figures, review and final editing. All authors contributed to the article and approved the submitted version.

## Conflict of interest

The authors declare that the research was conducted in the absence of any commercial or financial relationships that could be construed as a potential conflict of interest.

## Publisher’s note

All claims expressed in this article are solely those of the authors and do not necessarily represent those of their affiliated organizations, or those of the publisher, the editors and the reviewers. Any product that may be evaluated in this article, or claim that may be made by its manufacturer, is not guaranteed or endorsed by the publisher.

## Abbreviations

AChE: Acetylcholinesterase
AD: Alzheimer’s Disease
Apaf-1: Apoptotic Protease Activating Factor-1
APP: Amyloid Protein Precursor
BACE: Beta site Amyloid Precursor Protein Cleaving Enzyme
BAX: Bcl-2 Associated X-protein
BBB: Blood–Brain–Barrier
Bcl-2: B-cell Lymphoma 2
BCRP: Breast Cancer Resistance Protein
BDNF: Brain Derived Neurotrophic Factor
CAT: Catalase
CI: Cerebral Ischemia
COVID-19: Corona Virus Disease of 2019 (COVID-19)
COX-2: Cyclooxygenase 2
CYP2E1: Cytochrome P-450 2E1
DA: Dopamine
Dyn: Dynorphin
ER-β: Estrogen Receptor Beta
GDNF: Glial cell line-derived Neurotrophic Factor
GSH: Reduced L-glutathione
GSTA4-4: Glutathione S-transferase A4-4
HD: Huntington’s Disease
IFN-γ: Interferon gamma
IL-10: Interleukin 10
IL-1β: Interleukin 1 Beta
IL-6: Interleukin 6
iNOS: Inducible nitric oxide synthase
LD50: Lethal Dose 50
LDL-C: Low-density Lipoprotein Cholesterol
MAPK: Mitogen-activated Protein Kinase
MCT8: Monocarboxylate Transporter 8
MDA: malondialdehyde
MRP2: Multidrug Resistance-associated Protein 2
MS: Multiple sclerosis
NAFLD: Non-Alcoholic Fatty Liver Disease
NDDs: Neurodegenerative Disorders
NDs: Neurological Disorders
NE: Norepinephrine
NF-κB: Nuclear Factor Kappa B
NO: nitric oxide
PD: Parkinson’s Disease
P-gp: P-glycoprotein
SARS-CoV-2: Severe Acute Respiratory Syndrome Corona Virus 2
SOD: Superoxide dismutase
STAT-1: Signal Transducer and Activator of Transcription 1
T2D: Type 2 Diabetes
TLR4: Toll-like Receptor 4
TNF-α: Tumor Necrosis Factor Alpha
TNF-β: Tumor Necrosis Factor Beta
TrkB: Tropomyosin receptor kinase B
Treg: Regulatory T-cells
VMAT2: Vesicular Monoamine Transporter
WHO: World Health Organization
